# Why differentiate low molecular weight heparins for venous thromboembolism?

**DOI:** 10.1186/1477-9560-5-8

**Published:** 2007-06-19

**Authors:** Jawed Fareed, Jeanine M Walenga

**Affiliations:** 1 Loyola University Chicago, Stritch School of Medicine, Maywood, Illinois 60153 USA

The low molecular weight heparins (LMWHs) represent a group of antithrombotic and anticoagulant drugs which were initially developed for the prophylaxis of surgical thrombosis some 20 years ago. Today these drugs are used in expanded indication including venous thrombosis, cardiovascular disorders, thrombotic and ischemic strokes, cancer and neurodegenerative diseases. Because of the polytherapeutic actions these drugs can be differentiated from other older and newer antithrombotic drugs as stated in the following.

1. Differentiation of low molecular weight heparins from unfractionated heparins

2. Differentiation of commercially available low molecular weight heparins, which are produced by different methods.

3. Differentiation of the branded low molecular weight heparins from the generic versions of these drugs.

4. Differentiation of low molecular weight heparins from synthetic heparin derived oligosaccharides such as the pentasaccharides such as Arixtra and Idraparinux.

5. Differentiation of low molecular weight heparins from heparinoids and GAG mixtures such as the danaparoid, dermatan and heparans.

6. Differentiation of low molecular weight heparins from synthetic anti-Xa agents.

7. Differentiation of low molecular weight heparins from synthetic anti-IIa agents

8. Differentiation of low molecular weight heparins from oral anticoagulant drugs such as warfarin.

It is now clear that the clinical spectrum of the LMWHs is relatively broader from the different anticoagulant/antithrombotic drugs. Moreover, these agents have multiple interactions with plasmatic and cellular components and produce polytherapeutic effects. The synthetic monotherapeutic agents therefore will not have the same broad clinical profile as the LMWHs. It is important to differentiate the therapeutic effects of individual LMWH as each of these products is approved for specific indications. More recently dalteparin is approved for the treatment of cancer-associated thrombosis, an indication, which is solely approved for this product for this time. Therefore, it is important to differentiate these drugs and caution should be exercised for therapeutic and generic interchange.

Of all anticoagulants developed during the past 25 years, LMWHs have become the standard of care for the management of venous thromboembolism. Unlike unfractionated heparin (UFH), different brands of LMWHs can be differentiated biologically and clinically. Based on their different pharmacological and therapeutic profiles, LMWHs are largely considered by the experts and regulatory agencies such as the US FDA to be distinct drugs requiring indication and dosage specifications for each product. Therapeutic and generic interchanges are not recommended among different LMWHs, as this may compromise medical and surgical patient care [1]. The differences among the LMWHs are due to their molecular and structural properties.

LMWHs are a biological product derived from UFH obtained from animal tissue. In this regard, heparins are not drugs with one simple chemical structure, such as aspirin and statins. Heparins are a complex mixture of heterogeneous sugar chains. To produce LMWHs, the long chains of UFH are cleaved by different procedures so that the *average *molecular weight of the resulting heparin is of a lower molecular weight.

Beyond the *average *molecular weight, however, LMWHs can be further characterized [2-5]. Depending on the degradation processes (chemical β elimination [enoxaparin], enzymatic β elimination [tinzaparin], nitrous acid depolymerization [dalteparin], oxidative cleavage [ardeparin]) different compounds are generated. If the individual heparin chains that can contain 2–40 sugar units are identified, different proportions are present within each LMWH. Chemical modifications are also made to the saccharide chains, either at the cleavage endpoint or within the chain. Specific structural attributes that may or may not be induced by each degradation process include microchemical changes (e.g., addition or deletion of sulfate and acetyl groups), charge density, double-bond formation, formation of anhydro-manno or anhyro-gluco groupings, and the presence of 5-membered rings. For enoxaparin, approximately 30% of its molecules cannot be characterized by direct analysis. Thus, there are likely to be additional structural differences that would cause differentiation among LMWHs.

Due to the average molecular-weight difference of LMWHs from UFH, LMWHs generally have a lesser ability to inhibit thrombin (anti-FIIa activity), increased bioavailability, and decreased clearance rate. However, the specific chemical or structural features of each LMWH further translate into individualized biological and pharmacological characteristics of each LMWH. The chemical features contribute to differential binding affinities to antithrombin, von Willebrand's factor, growth factors, and other plasma proteins, interactions with platelets, leucocytes, vascular endothelial cells (release of tissue factor protein inhibitor is one example), and other biological components. Thus, beside the anticoagulant action, antithrombotic, anti-inflammatory, antiproliferative, and immunological actions are affected by the structural attributes of a LMWH. Only 20% of the components of heparins are anticoagulant in nature. The other 80% exhibit multiple biological actions that contribute to the overall effects of LMWHs, although this is not yet fully understood.

These variable biological interactions are reflected in differing pharmacological characteristics, including absorption, elimination half-life, and renal clearance [2-5]. Both the biological and pharmacological differences impact on the clinical safety and efficacy of each LMWH. The clinical performance of enoxaparin, dalteparin, and nadroparin has been differentiated in various settings [1,6]. Levels of evidence also exist relating to the variable effects of different LMWHs on specific patient populations, e.g., patients with renal impairment. These clinical differences among LMWHs would be more obvious with the higher dosages and extended treatment modalities that are being developed.

Therefore, LMWHs are complex biological drug products of comparable molecular weight, but with individual biological, pharmacological, and clinical characteristics. Potency adjustments of these complex drugs, based solely on anti-FXa and anti-FIIa do not minimize the variations between the LMWHs. Similarly, new antithrombotic agents [7,8] should be differentiated from the LMWHs on the basis of product quality, mechanism of drug antithrombotic activity, pharmacological behavior, and clinical safety and efficacy.

The LMWHs can also be differentiated from oral anticoagulant drugs such as warfarin. More importantly, being a small molecule warfarin is capable of passing the placental barriers and can not be used in pregnancy. While definitive clinical trials validating the use of LMWHs in pregnancy are not available at this time, this is one indication where these drugs may be very useful. The newly developed anti-IIa drugs such as dabigatran and anti-Xa drugs such as epixiban are also capable of passing the placental barrier and therefore cannot be used in this indication. Furthermore, all of these drugs are small molecular weight peptidomimetics which may exhibit some hemodynamic effects and modulation of hepatic enzymes. As such, the anti-IIa and anti-Xa drugs are also not the same and can be differentiated within their own group.

Venous thrombosis regardless of its origin is a complex pathophysiologic process. While tissue factor, coagulation proteases and their inhibitors contribute to the overall pathogenesis of thrombosis several additional factors play an important role in the overall clinical picture of this syndrome. Thus, single target drugs are of limited value for the overall management of VTE. Moreover the toxicity of these agents is not understood at this time. Therefore, one should be cautious in recommending these drugs as potential replacement for LMWH and oral anticoagulant drugs.

In summary, despite the development of several newer mono-therapeutic agents such as the anti-FXa, anti-FIIa, and recombinant protein inhibitors, the LMWHs remain the standard of care for venous thromboembolism management. Thrombosis is a polypathologic syndrome and can be best managed by poly-therapeutic drugs such as LMWHs. Because of the structural and functional differences among the currently available LMWHs the therapeutic interchange of these drugs is not recommended [9]. Moreover, because of the lack of proper guidelines for the acceptance of the generic versions of LMWHs the generic interchange is not recommended until proper guidelines and biologic equivalence is established.
